# Role of Cardiac Myocytes Heart Fatty Acid Binding Protein Depletion (H-FABP) in Early Myocardial Infarction in Human Heart (Autopsy Study)

**DOI:** 10.3889/oamjms.2016.018

**Published:** 2016-01-21

**Authors:** Amany Shabaiek, Nour El-Hoda Ismael, Samar Elsheikh, Hebat Allah Amin

**Affiliations:** 1 *Egyptian Forensic Medicine Authority, Pathology Department, Cairo, Egypt*; 2 *Faculty of Medicine, Kasr El- Aini, Pathology Department, Cairo, Egypt*

**Keywords:** detection myocardial infarction, H-FABP, immunohistochemistry, MI, sudden cardiac death

## Abstract

**BACKGROUND:**

Many immunohistochemical markers have been used in the postmortem detection of early myocardial infarction.

**AIM:**

In the present study we examined the role of Heart-type fatty acid binding protein (H-FABP), in the detection of early myocardial infarction.

**MATERIAL AND METHODS:**

We obtained samples from 40 human autopsy hearts with/without histopathological signs of ischemia.

**RESULTS:**

All cases of definite and probable myocardial infarction showed a well-defined area of H-FABP depletion. All of the control cases showed strong H-FABP expression, except two markedly autolysed myocardial samples that showed affected antigenicity.

**CONCLUSION:**

Thus, we suggest H-FABP as being one of the valuable tools facing the problem of postmortem detection of early myocardial infarction/ischemia, but not in autolysis.

## Introduction

Myocardial infarction (MI) is the most frequent diagnosis made in the majority of sudden deaths subjected to clinical and medico legal autopsies [1]. The problem is to recognize an infarct that has occurred within a very short time [2].

It is impossible to get the histological diagnosis of early myocardial infarction as a cause of sudden death in routine H&E stained sections, as it is unlikely to find special changes for myocardial infarction aged less than 6 hours [3, 4].

Thus, in the current autopsy practice, there is a great need for more sensitive diagnostic methods for the postmortem diagnosis of early myocardial damage [5].

Also, we need further studies on the postmortem stability of immunohistochemical markers and the regularity of their expression alteration in different postmortem intervals to define the role of autolysis [6].

Heart-type fatty acid binding protein (H-FABP) is a low molecular weight protein (14-15kDa), which is abundant in the cytoplasm of myocardial cells. As myocardial cell membrane is damaged by ischemia, H-FABP leaks to the extracellular space and enters the blood circulation very easily and quickly due to its small size and water solubility [7].

Therefore, loss of H-FABP in myocardial ischemic cells could occur as early as 15 minutes, and as the ischemia intervals prolonged, the depletion areas increase gradually in myocardial cells. The deletion areas of H-FABP reach a peak after four hours of myocardial ischemia [8].

The primary results suggest that H-FABP staining can detect very early ischemic damages in human myocardium [8, 9].

In the present study we examined the role of Heart-type fatty acid binding protein (H-FABP), in the detection of early myocardial infarction.

## Material and Methods

### Tissues

The study included 40 hearts from autopsy cases; with/without evidence of coronary artery disease (CAD). CAD cases included advanced or complicated lesions.

Tissue sections, from the myocardia and the corresponding coronary artery supply were obtained. They were fixed in 10% neutral-buffered formalin and embedded in paraffin. H&E stained sections are obtained and examined histologically.

Cases were grouped according to the histological findings as GI, GII, and GIII according to the Lodge Patch histological criteria for MI [10]:

1. Group I: includes 15 hearts represented cases showing signs of old ischaemia (fibrosis, granulation tissue and established infarction).
2. Group II: includes 18 cases represented cases showing either signs of recent ischemia (coagulative necrosis, contraction band necrosis, etc.) or with evidence of advanced or complicated CAD (severe stenosis, complicated atheroma or recent thrombus).
3. Group III: includes 7 cases showing no signs of ischemia, two of them showed severe autolytic changes which were selected to assess the expression of H-FABP in autolysis.

Death/Autopsy time ranged between 12-24 hours in all examined cases, except for the two autolysed control cases where death/autopsy time exceeded 60 hours.

Finally, we obtained 40 sections of myocardial tissue from the 40 cases for immunohistochemical staining by H-FABP.

### Immunohistochemistry

Immunohistochemical staining with H-FAB is performed using a standard avidin–biotin–peroxidase system.

Approximately 4 µm thick histologic sections were deparaffinated in xylene and alcohol, rehydrated in distilled water for 5 minutes, and then washed in PBS for 5 minutes. To reveal the antigens, they were pre-treated by using the proteolytic enzyme proteinase K and then washed in PBS for 5 minutes.

The primary monoclonal antibody to H-FABP (Aviscera Bioscience, USA) was diluted to a concentration of the antibody 1:250 (the optimal concentration supplied by the manufacturer) and incubated for 60 minutes at 37°C, then washed in PBS for 5 minutes.

Secondary antibody was applied for 60 minutes (DAKO, Denmark), followed by rinsing with PBS. The reaction was visualized with DAB chromogen (DAKO, Denmark). The slides were counterstained with diluted hematoxylin.

Each slide has its built in negative and positive controls for H-FABP. Areas with definitive signs of ischaemia by hematoxylin and eosin (H&E) acted as negative internal control, whereas non- ischemic regions of myocardium are expected to be positive internal control.

### Microscopic Evaluation

The amount and extent of depletion of the cell marker is recorded. Loss of myocardial staining of H-FABP in the three groups was recorded. The obtained results were scored from (0) to (-3) according to the percent of myocardial fibers showing depletion of H-FABP staining: Score 0: no loss of H-FABP staining, Score -1: loss in less than 25% of fibers, Score -2: loss in 25-50% of fibers, Score -3: loss in >50% of fibers [11].

## Results

The results are summarized in the Table 1.

**Table 1:** immune reactivity of H-FABP in all study groups. Data are presented as number (percent)

		H-FABP Score	Frequency	Percent	Valid Percent	Cumulative Percent
Valid	Group III	0	5	71.4	71.4	71.4

		-2	1	14.3	14.3	85.7

		-3	1	14.3	14.3	100.0

		Total	7	100	100	

	Group II	-1	2	11.1	11.1	11.1

		-2	7	38.9	38.9	50.0

		-3	9	50.0	50.0	100.0

		Total	18	100.0	100.0	

	Group I	-1	2	13.3	13.3	13.3

		-2	6	40.0	40.0	53.3

		-3	7	46.7	46.7	100.0

		Total	15	100	100	

**Figure 1:**
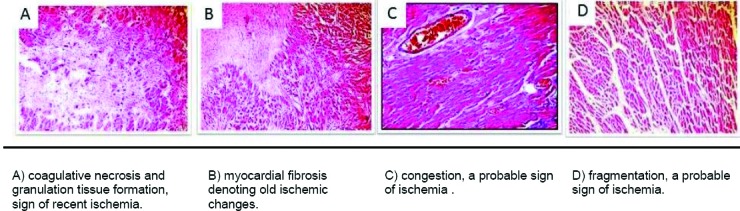
*Myocardial ischemia with Hematoxylin and eosin stain (x 200)*.

**Figure 2:**
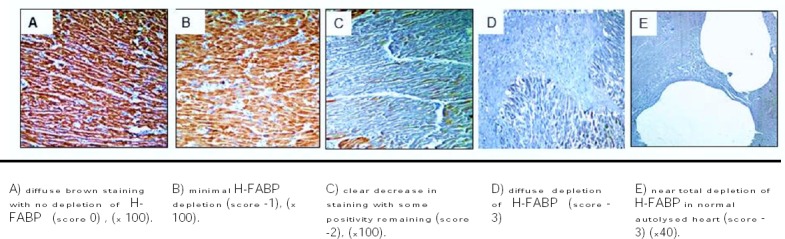
*H-FABP immunostaining with hematoxylin counterstain*.

Myocardial ischemia with Hematoxylin and eosin stain (x 200) is shown in Figure 1, and H-FABP immunostaining with hematoxylin counterstain is shown in Figure 2.

Group I [Figure 1B]; all cases showed variable degrees of H-FABP depletion. 7 out of 15 cases showed near total loss of cytoplasmic brown staining (score -3) [Figure 2D], 6 cases showed focal areas with loss of cytoplasmic brown staining (-2), and 2 cases showed minimal loss of staining (score -1) in myocardial areas surrounding the known ischemic areas.

Group II [Figures 1A, 1C and 1D]; all cases showed variable degrees of H-FABP depletion. 9 out of 18 cases showed near total loss of cytoplasmic brown cytoplasmic staining (score -3), 7 cases showed focal areas of loss of cytoplasmic brown staining (-2) [Figure 2C], and 2 cases showed minimal loss in staining (score -1) [Figure 2B].

Group III; 5 of the selected 7 cases showed strong brown cytoplasmic staining with no loss (score 0), denoting the integrity of the myocardium [Figure 2A]. The remaining two cases were selected with advanced autolytic changes to assess the efficacy of the H-FABP on autolysis. One of them showed near total loss (score -3) [Figure 2E] and the other showed wide areas of loss (score -2).

## Discussion

Histopathological examination of the heart must be performed as a part of routine autopsy as to clarify the cause of death, especially in non-traumatic deaths in which the autopsy didn’t reveal any confessant non-cardiac cause of death [12].

Many studies have proved the usefulness of variable immunohistochemical markers (especially Troponin, myoglobin, C5a, S100a1 and desmin) in the diagnosis of early myocardial injury has been promoted because most of them can be visible very early after the beginning of the symptoms (chest pain and angina attacks) [13-16].

However, very few studies have indicated the usefulness of H-FABP in cardiology for detection of acute myocardial infarction (AMI) in both human and animal models [7, 8].

On studying H-FABP immunostaining, all group I cases with old myocardial infarction, showed H-FABP depletion in the infarcted areas.

These results are consistent with the results obtained by Meng et al., 2006, who recognized H-FABP depletion in infarcted area in the human myocardia [9]. They are also comparable to the results given by Ming et al 2004, where all 7 cases with definite infarcted area, showed significant or total loss of H-FABP in fibrous tissue (confirmative infarcted area) [8].

We also noticed variable degrees of H-FABP depletion in the myocardial fibers surrounding the infarcted areas that appeared normal in the H&E stained slides. This was rather more remarkable than the results of Ming et al., 2004 who declared that these apparently normal cardiomyocytes were brown mixed with some faintly immunostained cardiomyocytes [8].

An example from this group was a heart with left ventricular concentric hypertrophy. Microscopic examination revealed severe stenosis in his left circumflex artery and minimal foci of myocardial fibrosis in the lateral wall of left ventricle. H-FABP staining of the section selected from and around the fibrotic foci revealed wider areas of ischemia - loss (-2) - around fibrotic foci denoting more pronounced ischemia, which could only be detected by the antibody, giving the impression of its high sensitivity.

Selected group II cases, with probable signs of ischaemia had coronary stenosis and their complication showed loss of cytoplasmic staining by various degrees ranging from minimal depletion (-1) and up to near total depletion (-3). Meng et al, 2006, in their study, noticed H-FABP depletion in areas of suspected early myocardial infarction that showed normal H&E staining [9].

Worth mentioning, one of group II cases belonging to a male in his 5^th^ decade of life. He died in custody half an hour after exposure to a homicidal attempt by strangulation by another prisoner. On autopsy, there were only bruises on neck side. Examination of the heart revealed no gross myocardial abnormalities. Microscopic examination revealed severe stenosis of the left anterior descending coronary artery with rupture in arterial wall atheromatous plaque complicated by recent thrombus formation. Myocardium showed only congestion and an area of fragmentation by H&E staining. However; immunohistochemical examination of the myocardium revealed areas of H-FABP depletion (score -2) documenting early ischemic changes.

Acute coronary syndrome found in this case led to death with the subsequent myocardial ischemia only detected by Immunohistochemical staining [6].

On examining group III cases - control positive group- five out of the seven cases showed diffuse cytoplasmic H-FABP immunoreactivity exhibited in the cardiac myocytes (score 0). In comparison with the study performed by Meng et al., 2006, cases with no signs of ischemia, showed weak, but diffuse staining [8].

The remaining two cases that showed depletion in this group were two autolysed hearts from two putrefied female cadavers, with no evidence of ischemia. Estimated time of death exceeded 60 hours. Both cases showed H-FABP depletion, which reflects that H-FABP immunoreactivity in this study was markedly affected by autolysis.

This is contradictory to the results recorded by Ming and Meng, 2004. They stated that the efficacy of H-FABP for detection of ischemic cardiomyocyte lesions was not reduced by autolysis, even in cases where death autopsy time extended as long as 60 hours after death on human hearts [8].

We can summarize that H-FABP may be a useful marker in the early detection of AMI as well as the ongoing ischemia in areas surrounding well established infarctions in human hearts which can’t be detected using routine H&E stain, as well as the ongoing ischemia in areas surrounding well established infarctions.

However; it is not the marker of choice that can be used in the detection of early myocardial ischemia in advanced autolytic changes - despite its sensitivity generally. Other antibodies that can withstand autolysis as troponin, myoglobin and desmin can do that role instead [5, 11, 17].

Many factors are needed for well-established confident diagnosis besides the immunohisto-chemistry. We should correlate with the history, clinical data and case circumstances and their insufficiency may act as an obstacle in achieving satisfactory results that we are aiming at. In forensic practice, we can’t give opinion depending on single factor, there are many factors should be put together to get out at the end by reliable confident decision, and every case in our practice has its unique characteristics, circumstances and findings that make it an indivisible standalone entity.
